# Objective measurement of head movement differences in children with and without autism spectrum disorder

**DOI:** 10.1186/s13229-018-0198-4

**Published:** 2018-02-27

**Authors:** Katherine B. Martin, Zakia Hammal, Gang Ren, Jeffrey F. Cohn, Justine Cassell, Mitsunori Ogihara, Jennifer C. Britton, Anibal Gutierrez, Daniel S. Messinger

**Affiliations:** 1 Department of Psychology, University of Miami, 5665 Ponce de Leon Blvd, Coral Gables, FL 33146 USA; 2 Robotics Institute, Carnegie Mellon University, 5000 Forbes Ave, Pittsburgh, PA 15213 USA; 3 Center for Computational Science, University of Miami, 1320 S Dixie Hwy, Miami, FL 33146 USA; 4 Department of Psychology, University of Pittsburgh, 210 S. Bouquet St., Pittsburgh, PA 15260 USA; 5 Human Computer Interaction, Carnegie Mellon University, 5000 Forbes Avenue, Pittsburgh, PA 15213 USA; 6 Department of Computer Science, University of Miami, 1365 Memorial Drive, Coral Gables, FL 33146 USA

**Keywords:** Head movement, Motor movement, Autism spectrum disorder, Social processing

## Abstract

**Background:**

Deficits in motor movement in children with autism spectrum disorder (ASD) have typically been characterized qualitatively by human observers. Although clinicians have noted the importance of atypical head positioning (e.g. social peering and repetitive head banging) when diagnosing children with ASD, a quantitative understanding of head movement in ASD is lacking. Here, we conduct a quantitative comparison of head movement dynamics in children with and without ASD using automated, person-independent computer-vision based head tracking (Zface). Because children with ASD often exhibit preferential attention to nonsocial versus social stimuli, we investigated whether children with and without ASD differed in their head movement dynamics depending on stimulus sociality.

**Methods:**

The current study examined differences in head movement dynamics in children with (*n* = 21) and without ASD (*n* = 21). Children were video-recorded while watching a 16-min video of social and nonsocial stimuli. Three dimensions of rigid head movement—pitch (head nods), yaw (head turns), and roll (lateral head inclinations)—were tracked using Zface. The root mean square of pitch, yaw, and roll was calculated to index the magnitude of head angular displacement (quantity of head movement) and angular velocity (speed).

**Results:**

Compared with children without ASD, children with ASD exhibited greater yaw displacement, indicating greater head turning, and greater velocity of yaw and roll, indicating faster head turning and inclination. Follow-up analyses indicated that differences in head movement dynamics were specific to the social rather than the nonsocial stimulus condition.

**Conclusions:**

Head movement dynamics (displacement and velocity) were greater in children with ASD than in children without ASD, providing a quantitative foundation for previous clinical reports. Head movement differences were evident in lateral (yaw and roll) but not vertical (pitch) movement and were specific to a social rather than nonsocial condition. When presented with social stimuli, children with ASD had higher levels of head movement and moved their heads more quickly than children without ASD. Children with ASD may use head movement to modulate their perception of social scenes.

**Electronic supplementary material:**

The online version of this article (10.1186/s13229-018-0198-4) contains supplementary material, which is available to authorized users.

## Background

Autism spectrum disorder (ASD) is characterized by persistent impairments in social interaction and communication, as well as repetitive and stereotyped behaviors [1]. Previous research has identified deficits in motor development [2] and higher levels of motor stereotypies in children with ASD than children without ASD [3]. Atypical movement patterns, such as abnormalities in eye contact and body posture, and motor stereotypies are used in the evaluation of ASD, but little attention has focused on characterizing these motor differences through automated, objective measurement [1, 3, 4]. The current study examined whether head movement dynamics differentiated children with and without ASD, and contrasted head movement while watching video of nonsocial and social stimuli.

While movement stereotypies are common in typically developing infants, they decrease rapidly over the first 2 years of life [3]. Atypical head movements in young children have garnered little attention, even though this stereotypy is clinically viewed as highly suggestive of ASD [3, 5, 6]. Descriptively, clinicians have noted that children with ASD exhibit atypical head movements as they stare at their fingers or objects closely from a “strange angle” [3], repetitively peer at objects “from the side” [7], and examine objects from “odd angles or peripheral vision” [8]. Goldman et al. [3] found that this stereotypy is rare, but seemingly specific to children with ASD.

Head movement stereotypy may be an adaptive strategy that facilitates perception or social communication [9, 10]. Turning away from over-stimulating stimuli often marks a child’s need to self-regulate [11]. By engaging in head movement stereotypies or similar movements, individuals with ASD may be regulating incoming visual and social information that is perceived as over-arousing [9].

On the other hand, atypical head movements in children with ASD may contribute to the social impairments that characterize children with ASD. Motor movement is crucial for verbal and nonverbal communication, formation of friendships, and the maintenance of social interactions. Head nods and turns, for example, serve to influence turn-taking between social partners [12]. In successful social interactions, motor movements must be initiated and coordinated [13] as typical motor control functions link the perception of other’s actions and one’s own actions [14]. Motor delays in ASD, such as the inability to coordinate functional head and arm movements, may prevent head turning in response to one’s name and gaze following, and contribute to failures to engage in gestural nonverbal communication such as joint attention [11]. Better quantification of these motor movements will further our understanding of their role in the development of ASD.

Motor movement in ASD has typically been assessed descriptively via parent report and trained human observers. While parents have opportunities to observe their children in multiple contexts, their reports are prone to bias [4, 15]. Coding schemes of motor movement and stereotypies conducted by trained observers are frequently study-specific and receive little or no independent validation [3, 16]. In response to the limitations of qualitative efforts, automated measurement has been used to objectively document atypical motor movement and stereotypies [3, 4, 16–18]. ASD is associated with atypical gait in toddlers and children [19–22], reduced postural stability in children [23–26], and increased repetitive and stereotypic behaviors in children [3, 27, 28]. A recent meta-analysis revealed that motor impairments in movement preparation, upper extremity motor function, and gait were significantly more pronounced in individuals with ASD than individuals without ASD [4].

Automated measurement and machine-learning algorithms have been used to examine motor movements to both enhance clinical assessment [29, 30] and to elucidate the mechanisms and heterogeneity of ASD [22, 31–33]. Machine learning algorithms have successfully distinguished children with severe ASD (age 2–4 years) from children without ASD during a reach-to-grasp task [29]. Machine learning analysis of motor patterns of children playing with smart tablet computers correctly identified children with ASD from children without ASD [30]. Children with ASD contacted the table with greater force, had different distributions of force within a gesture, and displayed faster and larger movements than children without ASD [30, 32]**.**

An initial report on postural sway examined head movement differences between children with and without ASD. Children with ASD exhibited greater head movement and sway while standing than children without ASD, and both groups reduced their postural sway during performance of a nonsocial task [23]. However, with the exception of postural sway tasks [23], investigations of motor movement have not focused on head movements in children with and without ASD. Taken together, previous research supports the importance of head movement atypicalities in ASD and suggests they warrant further exploration.

### Current study

We conducted a quantitative comparison of head movement dynamics in children with and without ASD, matched on mental age, between 2.5- and 6.5-years-old, using an automated head tracking system. In lieu of subjective, manual coding, automated tracking provided objective measurement to quantify differences in head movement dynamics. We hypothesized that children with ASD would exhibit greater and more rapid head movement than children without ASD. As children with ASD typically exhibit preferential attention to nonsocial versus social stimuli [34–36], we conducted an *a priori* analysis to ascertain whether differences in head movement dynamics between children with and without ASD varied by social and nonsocial stimulus presentation.

## Methods

### Participants

Participants were 2.5–6.5-year-old children (*mean* = 4.72 years, SD = 1.14 years, *range* = 4.25 years) with (*n* = 21) and without (*n* = 21) ASD. Children with ASD were the older siblings of infants recruited from a longitudinal study of high-risk development. Children without ASD were typically developing children, with no reported risks or diagnoses at the time of study, and were recruited from a longitudinal study of high-risk development and from the community, through recruitment flyers. Children were excluded from the study if they had a gestational age below 37 weeks or major birth complications. Parents were reimbursed $50 for their child’s participation in the study. Recruitment and procedures were approved by the University’s Internal Review Board and written, parental consent was obtained before participation.

### Measures and procedure

Clinical diagnosis of ASD or the absence of ASD was determined at study entry. The Autism Diagnostic Observation Schedule [37] and Autism Diagnostic Interview-Revised [38] were used to inform the DSM-IV-based best estimate diagnosis from a licensed psychologist, who was unfamiliar with the child’s previous diagnosis. To assess children’s mental age, children were administered with either the Wechsler Preschool and Primary Scale of Intelligence (*n* = 33; WPPSI-III, [39]) or the Mullen Scales of Early Learning (*n* = 6; Mullen, [40]). The Mullen was typically administered when children were 37 months of age or younger. Except for two 36-month-olds (1 ASD, 1 No ASD), the WPPSI was administered when childern were older than 37 months. Three children (2 ASD, 1 No ASD) did not receive a cognitive assessment. Groups were comparable on the assessments administered, χ^2^(2) = 1.27, *p* = .53.

Groups were matched a priori on mental age [41]. Groups did not differ on chronological age, *F* (1,41) = 4.00, *p* > .05; mental age, *F* (1,38) = .007, *p* > .05 (Table 1); or gender, Fisher’s exact test *p* = .58 (Table 2).

**Table 1:** Chronological age and mental age by ASD group

		N	Mean	SD
Age at visit (months)	No ASD	21	51.23	15.35
	ASD	21	60.80	16.52
Mental age (months)	No ASD	20	54.58	14.59
	ASD	19	54.08	22.95

Children with ASD did not differ from children without ASD on chronological or mental age

**Table 2:** Gender by ASD group

	Males	Females
No ASD	14	7
ASD	17	4

Children with ASD did not differ from children without ASD on gender

**Fig. 1:**
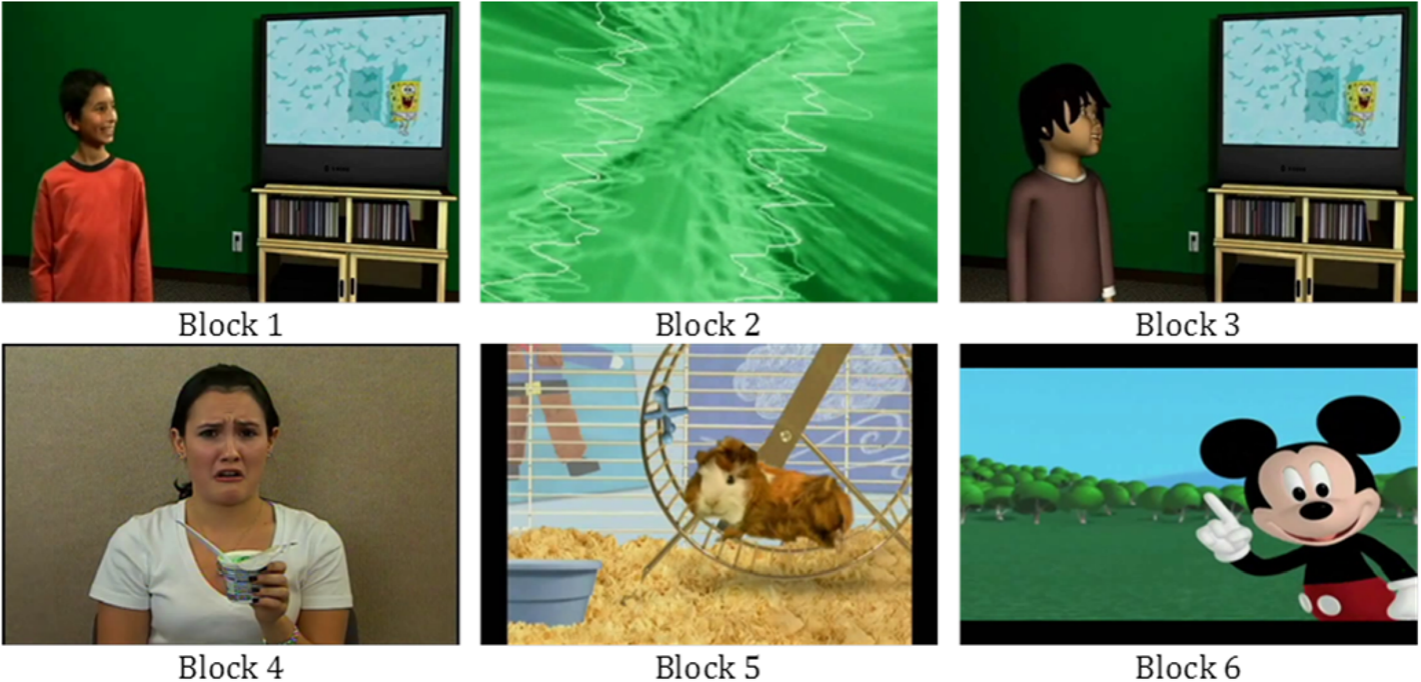
Stimuli presentation by video. The 16-min video consisted of social and nonsocial stimuli, designed to elicit joint-attention and emotion expression in young children

Children were seated approximately 65 cm in front of a 19-in. video monitor. They were asked to watch a short video, while a camera positioned on top of the monitor recorded their face and upper body at 29.971 frames/s. The protocol consisted of a 16-min video, composed of both social and nonsocial stimuli. The monitor displayed six videos of stimuli designed to elicit joint-attention and emotion expression in children. Video 1 was a 3-min social stimulus presentation of an actual boy pointing in a virtual environment to a side television of an animated character (SpongeBob), which was designed to elicit looks from the boy to the television (joint attention). Video 2 was a 2-min presentation of a non-social, audio-visual screensaver. Video 3 was a 3-min social stimulus presentation of an animated boy pointing in a virtual environment to a side television of an animated character (SpongeBob), which was designed to elicit looks from the boy to the television (joint attention). Video 4 was a social, 6-min emotion-eliciting story of a birthday party told by a woman. Video 5 was a social, 1-min Wonder Pets cartoon clip, and video 6 was a social, 1-min Mickey Mouse cartoon clip (Fig. 1).

Based on an a priori hypothesis, video 2 served as the nonsocial stimulus and the first 2-min of video 4 served as the social stimulus (the same pattern of results was observed when analyzing the full 6-min of video 4). Other videos contained a mixture of actual and animated figures and were not appropriate for sociality contrasts.

### Head tracking

To quantify head movement dynamics, a fully automatic, person-independent computer-vision algorithm was used to track pitch, yaw, and roll of head movement (http://zface.org/, Zface, [42]). For each video frame, the algorithm registered a dense 3D face shape in real-time. This was accomplished using a fast cascade regression framework trained on high-resolution 3D face-scans of posed and spontaneous face and head motion. Zface was computationally efficient but delivered high precision tracking. Experimental findings strongly support the validity of real-time, 3D registration and reconstruction from 2D video [42]. Compared to 10 other computer-vision based approaches for head tracking, Zface achieved the lowest absolute angular error for head pitch and the second lowest angular error for yaw (2.66 and 3.93 degrees, respectively) [43].

**Fig. 2:**
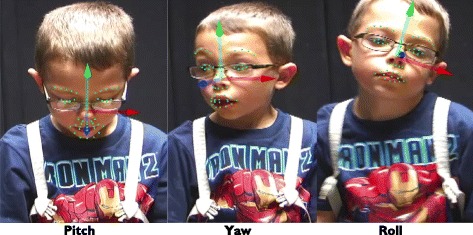
Head orientation. The 3° of rigid head movement (pitch, yaw, and roll) are indexed above by the x, y, and z arrows. The green arrow indexes pitch, the blue arrow indexes yaw, and the red arrow indexes roll

For each video frame, the algorithm outputted 3° of rigid head movement—pitch (vertical movement; head nods), yaw (horizontal movement; head turns), and roll (lateral head inclinations toward the shoulder) (Fig. 2) or a failure message when a frame could not be tracked (see Table 3 for the range of pitch, yaw, and roll).

**Table 3:** Range of pitch, yaw, and roll

	Minimum (radians)	Maximum (radians)	Minimum (degrees)	Maximum (degrees)
Pitch	− .75	1.16	42.97 down	66.46 up
Yaw	− .85	.84	48.70 left	48.13 right
Roll	− 1.05	1.13	60.16 left shoulder	64.74 right shoulder

17.4% of the frames could not be tracked, which is comparable with previous work in this area [44]. Several conditions contributed to tracking failure, including self-occlusion (hands on the face), extreme head movement, and location change (e.g., i.e., child moved out of the frame). Proportions of successfully tracked frames were examined for ASD group differences.

### Data reduction

To ensure that missing data would not bias measurements, head movement dynamics were measured separately for each consecutively tracked segment (epoch). Epochs were defined as successfully tracked consecutive frames within a video (mean epoch length = 577.35 frames, at 29.971 frames per second). A 2 (group) by 6 (video) repeated-measures ANOVA indicated that the number of epochs per video did not differ significantly between groups, *F* (40) = 2.70, *p* = .11, Marginal Mean_ASD_ = 18.1, Marginal Mean_NonASD_ = 8.0. A 2 (group) by 6 (video) showed that the mean duration of an epoch also did not differ significantly between groups, *F* (40) = 1.89, *p* = .18, Marginal Mean_ASD_ = 747.98 frames/epoch, Marginal Mean_NonASD_ = 1049.04 frames/epoch (Table 4). Nevertheless, children with ASD tended to have more epochs of briefer duration than children without ASD.

**Table 4:** Number of epochs and mean epoch duration by ASD Group

		Marginal Mean	*F*	*p* value
Number of Epochs	No ASD	8.00	2.70	.11
	ASD	18.00		
Mean Epoch Duration (frames)	No ASD	1049.04	1.89	.18
	ASD	747.98		

Within each epoch, head movement dynamics were quantified with respect to the three principal axes of pitch, yaw, and roll. For each of these axes, angular displacement and angular velocity were calculated for each frame of video. Angular values in displacement and velocity of pitch, yaw, and roll were measured in radians and radians/frame, respectively. For pitch, yaw, and roll, angular displacement was calculated as the difference between each observed head angle value and the overall mean of head angle within each epoch. Similarly, for pitch, yaw, and roll, angular velocity was calculated as the temporal derivative of the angular displacement for each movement direction using the finite difference method (the location difference between successive video frames).

The root mean square (RMS) then was used to measure the magnitude of angular displacement and angular velocity of pitch, yaw, and roll, respectively [44–47]. The RMS value was calculated as the square root of the arithmetic mean of the squares of the original values, in our case the angular displacements and the angular velocities. To account for the varying lengths of epochs caused by untracked frames, the RMS value for each epoch was weighted by its epoch duration. These weighted values were averaged across epochs to obtain a normalized RMS value (nRMS; Eq. 1). The obtained nRMS for angular displacement and angular velocity for pitch, yaw, and roll are used in subsequent analyses and are referred to as angular displacement and angular velocity for simplicity.

1 $$ {\mathrm{nRMS}}_x=\sqrt{\frac{1}{n}\left({x}_1^2+{x}_2^2+\dots +{x}_n^2\right)} $$

where *x*^2^_1_…*x*^2^_*n*_ are the squared differences between the value of a frame and the mean value of frames within an epoch.

### Analytic approach

#### Preliminary analyses

A preliminary 2 (group) × 6 (video) repeated-measures analysis of variance (ANOVA) compared the proportion of successfully tracked frames by ASD group to determine whether children with and without ASD differed in levels of automated tracking.

#### ASD group differences

A second 2 (group) × 6 (video) repeated-measures ANOVA was used to test for differences between children with and without ASD in the angular displacement and angular velocity of pitch, yaw, and roll respectively. We hypothesized that children with ASD would exhibit greater angular displacement and angular velocity of pitch, yaw, and roll than children without ASD.

#### ASD group by stimulus type interaction

Planned contrasts were then used to test for the interaction between stimulus type (social versus nonsocial) and group (ASD versus no ASD). A 2 (group) × 2 (Nonsocial_Video2_ vs. Social_Video4_) repeated-measures ANOVAs examined whether children with and without ASD differed in pitch, yaw, and roll angular displacement and angular velocity separately between nonsocial (video 2) and social stimuli (video 4). All main analyses were then repeated covarying chronological age to determine the degree to which differences between the mental-age-matched groups might be due to chronological age. (Analyses of supplementary head movement variables, which yielded results similar to those outlined below, are found in Additional file 1.)

## Results

### Preliminary analyses

**Fig. 3:**
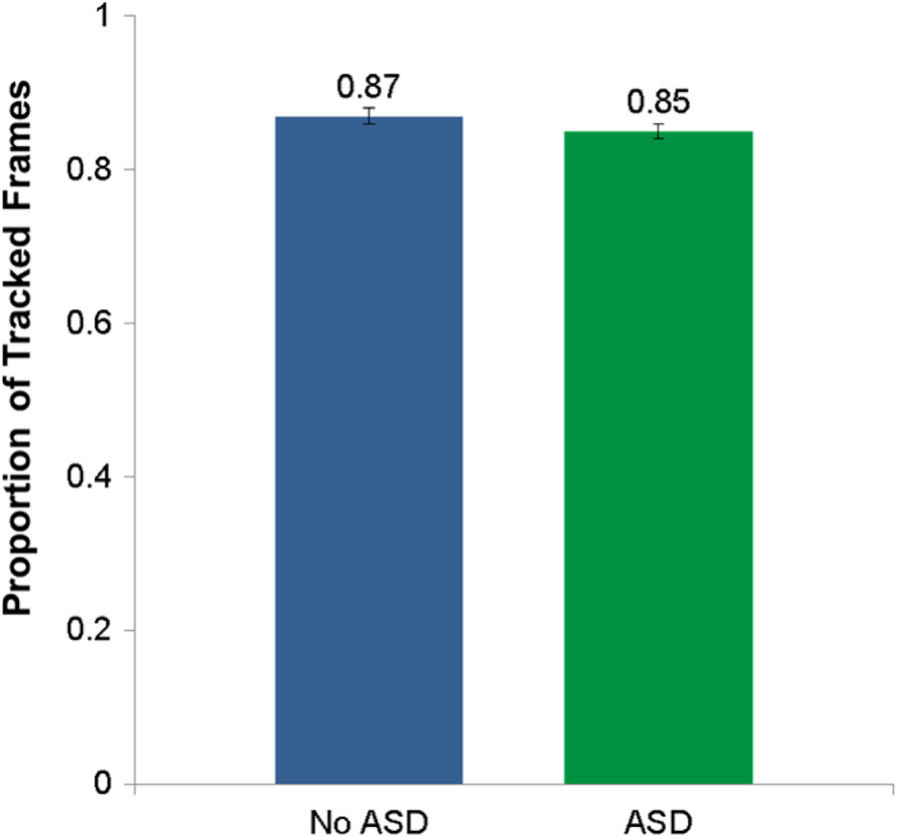
Proportion of tracked frames by group. Children with ASD did not differ in their proportion of frames successfully tracked by the automated software (Zface) than children without ASD. Overall, 85% of frames for children with ASD were tracked and 87% of frames for children without ASD were tracked. Error bars: ± 1 SEM

A one-way analysis of the proportion of successfully tracked frames over the entire course of the protocol revealed no group differences, *F* (39) = .08, *p* = .77, partial *η*^2^ = .003 (Fig. 3). A repeated-measures ANOVA indicated a main effect of video, *F* (3.58, 38) = 3.01, *p* = .03 *partial* η^2^ = .07, and no interaction of video by group, *F* (3.58, 38) = .15, *p* = .95, partial *η*^2^ = .004. There were no group differences in proportion of successfully tracked frames by video, *p*s > .69.

### ASD group differences

**Fig. 4:**
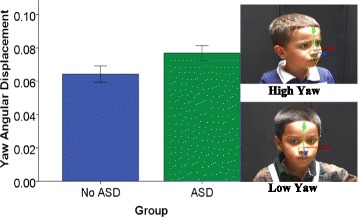
Between-group differences in yaw angular displacement. Children with ASD have greater yaw angular displacement than children without ASD. Note. Error bars: ± 1 SEM

For angular displacement, a 2 (group) × 6 (video) repeated-measures analysis of variance (ANOVA) revealed main effects of video for pitch and yaw. No significant interactions of video and group were found for the angular displacement of pitch, yaw, and roll. Children with ASD exhibited greater angular displacement of yaw than children without ASD, indicating greater head turning, *F* (1, 37) = 4.36, *p* = .04, partial *η*_p_^2^ = .11 (Fig. 4, Table 5). Children with ASD did not differ from children without ASD on pitch and roll angular displacement, *p*s > .05.

**Table 5:** Repeated-measures ANOVA of pitch, yaw, and roll

		df	*F*	*p*	*η*_p_^2^
ADis_Pitch	Video	3.74	3.21	.02*	.08
	Video*Group	3.74	0.40	.79	.01
	Group	1.00	1.81	.19	.05
ADis_Yaw	Video	4.85	3.97	<.01*	.10
	Video*Group	4.85	1.39	.23	.04
	Group	1.00	4.36	.04*	.11
ADis_ Roll	Video	2.71	0.42	.72	.01
	Video*Group	2.71	1.26	.29	.03
	Group	1.00	3.33	.08	.08
AVel_Pitch	Video	3.31	3.27	.02*	.08
	Video*Group	3.31	0.68	.58	.02
	Group	1.00	0.77	.39	.02
AVel_Yaw	Video	3.60	1.90	.12	.05
	Video*Group	3.60	0.57	.67	.02
	Group	1.00	4.01	.05*	.10
AVel_Roll	Video	3.46	2.58	.06	.07
	Video*Group	3.46	0.56	.67	.02
	Group	1.00	7.35	.01*	.17
					*p < .05

*ADis* angular displacement, *AVel* angular velocity

**Fig. 5:**
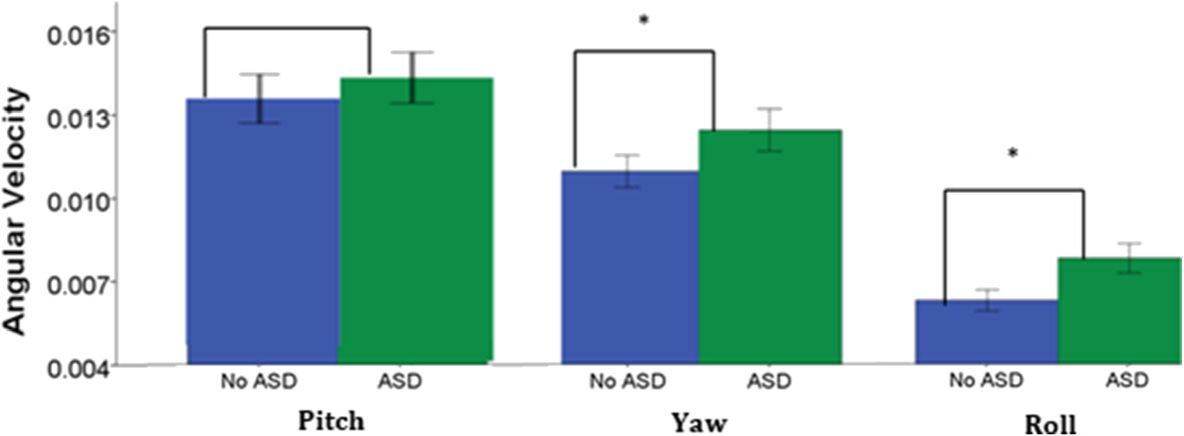
Between-group differences in yaw and roll angular velocity. Children with ASD had greater yaw and roll angular velocity than children without ASD. Note. Error bars: ± 1 SEM

For angular velocity, repeated-measures ANOVA revealed a main effect of video for pitch and roll. No significant interactions of video and group were found for angular velocity of yaw, pitch, and roll. Children with ASD exhibited greater angular velocity of yaw, *F* (1, 37) = 4.01, *p* = .050, partial *η*_p_^2^ = .10, and roll, *F* (1, 37) = 7.35, *p* = .010, partial *η*_p_^2^ = .17 than children without ASD, indicating greater head movement (Fig. 5, Table 5). Pitch angular velocity did not differ between children with and without ASD, *p* > .05.

### ASD group by stimulus type (social versus nonsocial video) interaction

**Fig. 6:**
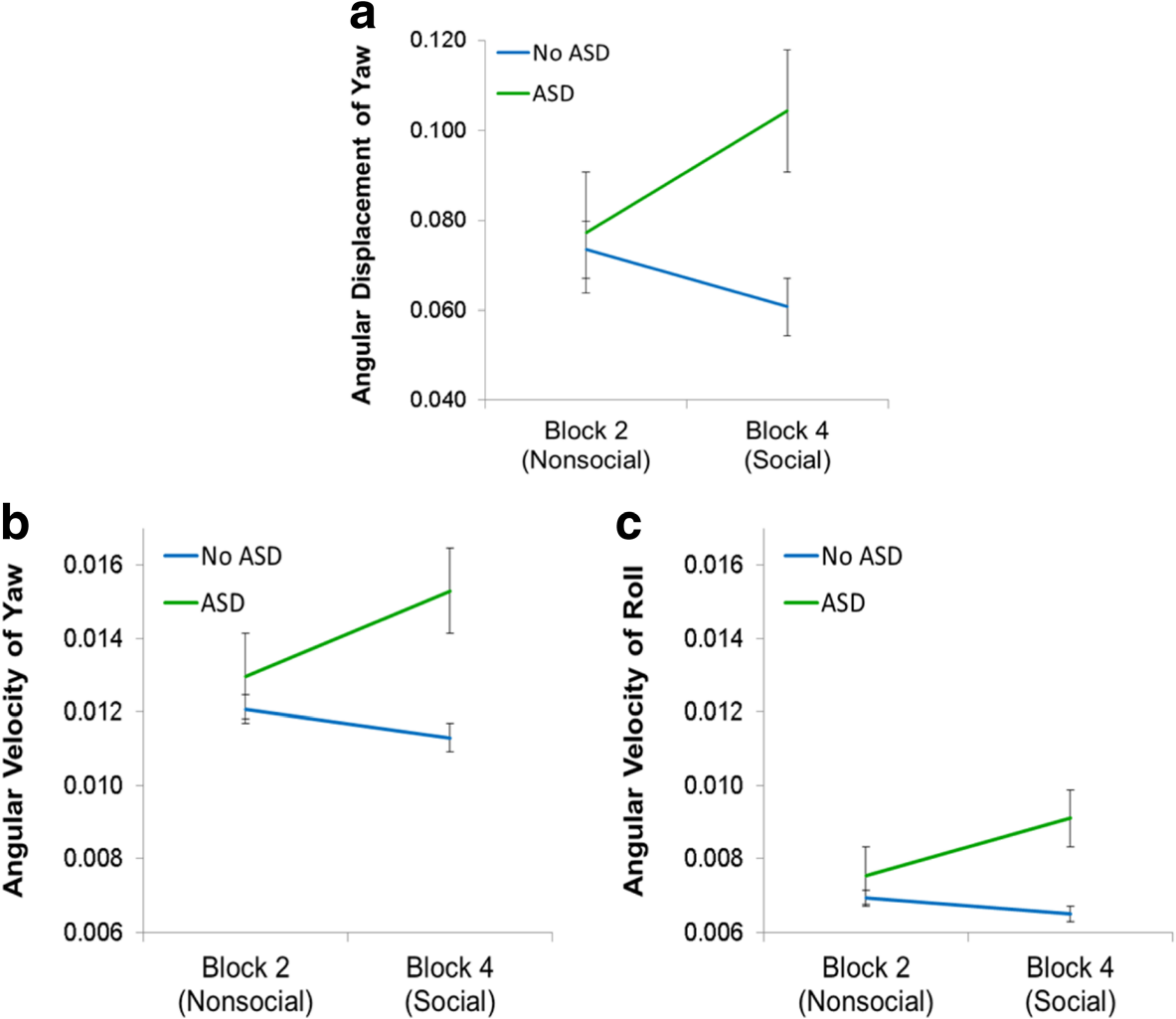
Video (nonsocial vs. social) by group interaction. Compared to children without ASD, children with ASD differed in angular displacement of yaw (**a**) and angular velocity of yaw (**b**) and roll (**c**) only during the social stimulus (video 4), but not the nonsocial stimulus (video 2). Error bars: ± 1 SEM

Planned contrasts revealed an interaction between video and group for yaw angular displacement, *F* (1,40) = 7.86, *p* < .01, *η*_p_^2^ = 16, and a significant between-subjects effect of group, *F* (1) = 5.99, *p* = .019, *η*_p_^2^ = .13 (Fig. 6). Children with ASD had greater angular displacement of yaw in the social video (video 4), than children without ASD, and did not differ in their angular displacement of yaw in the nonsocial video (video 2) than children without ASD. There were no interactions between video and group for angular displacement of pitch and roll, *p*s > .05.

For angular velocity of yaw, there was an interaction between video and group, *F* (1,40) = 8.35, *p* < .01, *η*_p_^2^ = .17, and a significant between-subjects effect of group, *F* (1,40) = 4.90, *p* = .033, *η*_p_^2^ = .11 (Fig. 6). There was also an interaction between video and group for angular velocity of roll *F* (1,40) = 4.27, *p* = .045, *η*_p_^2^ = .10, with a significant between-subjects effect of group, *F* (1,40) = 4.69, *p* = .036, *η*_p_^2^ = .11 (Fig. 6). Children with ASD had greater angular velocity of yaw and roll in video 4 (social video) than children without ASD and did not differ in their angular velocity of yaw and roll in video 2 (nonsocial video). There was no interaction between video and group for angular velocity of pitch, *p* > .05.

### Controlling for age

A 2 (group) × 6 (video) repeated-measures analysis of variance (ANOVA) was conducted with chronological age as a covariate. As when not considering this covariate, children with ASD exhibited greater angular displacement of yaw than children without ASD, indicating greater head turning, *F* (1, 36) = 5.36, *p* = .02, *η*_*p*_^*2*^ = .13. As when not considering the age covariate, children with ASD exhibited greater angular velocity of roll, *F* (1, 36) = 5.45, *p* = .02, *η*_p_^2^ = .13, than children without ASD, indicating greater head rolling motion. Unlike previous findings without age, children with ASD did not exhibit greater angular velocity of yaw, *F* (1, 36) = .73, *p* = .40, *η*_*p*_^*2*^ = .02 when controlling for chronological age. All other findings were unchanged.

The planned contrast models (social versus nonsocial video) were repeated with angular velocity including chronological age as a covariate. As in previous findings without age as a covariate, there was an interaction between video and group for angular velocity of yaw, *F* (1,39) = 4.83, *p* < .03, *η*_p_^2^ = .11, but there was no between subject’s effect of group, *F* (1,39) = 1.72, *p* = .20, *η*_p_^2^ = .04. Children with ASD had greater angular velocity of yaw in the social video (video 4) than children without ASD, and the two groups did not differ in the angular velocity of yaw and roll in the nonsocial video (video 2). Unlike previous analyses without age as a covariate, no interaction between group and video was found for angular velocity of roll when chronological age was included in the model, *F* (1,39) = 2.97, *p* = .09, *η*_p_^2^ = .07. All other findings were unchanged.

## Discussion

Using automated, objective measurement, we quantified differences in head movement dynamics between children with and without ASD, shedding light on head movement atypicalities previously described by clinicians. Children with ASD showed greater angular displacement of yaw and greater angular velocity of yaw and roll than children without ASD. Angular displacement is interpreted as head movement *quantity*, and angular velocity is interpreted as the *speed* of head movement. Thus, children with ASD exhibited greater head turning (yaw)—and turned their heads (yaw) and inclined their heads (roll) with greater speed—than children without ASD. Differences in head movement dynamics between children with and without ASD were specific to the presentation of a social stimulus. That is, children with ASD exhibited greater yaw angular displacement and yaw and roll angular velocity during presentation of the social stimulus than children without ASD.

Analyses were repeated including chronological age as a covariate—groups were matched a priori on mental age—to disentangle age and ASD differences [3, 48]. When controlling for chronological age, children with ASD continued to exhibit greater head turning (yaw) and inclined their heads (roll) with greater speed than children without ASD. When controlling for chronological age, differences in head movement dynamics between children with and without ASD remained specific to the presentation of a social stimulus for angular displacement of yaw and angular velocity of yaw, but not angular velocity of roll. Comparison of models, with and without statistical controls for chronological age, highlight angular displacement of yaw and angular velocity of yaw and roll as consistent signatures of ASD.

The current findings add to a small but growing body of literature utilizing automated measurement of body and head movement to objectively quantify the ASD phenotype [32, 49]. In a previous investigation, for example, 9-year-old children with ASD exhibited greater sway while standing in both the anterior-posterior (front-to-back) and medial-lateral (side-to-side) axes than did children without ASD, but sway was reduced during the search task, suggesting better movement control when pursuing a goal [23]. By contrast, we measured 3° of rigid head movement (pitch, yaw, and roll) from video-recordings of younger, seated children. Younger children with ASD exhibited greater head displacement and velocity in the horizontal (yaw) and lateral (roll) but not vertical (pitch) axes than children without ASD. These differences in displacement and velocity were specific to social stimuli presentation. Together, these findings suggest that nonsocial engagement constrains excess head movement dynamics in children with ASD, while spontaneous activity, particularly in reaction to social stimuli, is associated with increased head movement dynamics.

Children with ASD may use head movement as a way to modulate their sensory experience [50]. Previous primary research [4] and meta-analytic results of observational measures [51] indicate that infants and children with ASD displayed higher levels of motor impairments than infants and children without ASD. Motor impairments may constitute a core feature of ASD, a finding supported by the current studies comparisons of children with and without ASD [4, 32]. However, when head movement was compared during the presentation of social and nonsocial stimuli, head movement differences were specific to the presentation of social stimuli. Previous research using eye-tracking indicates that children with ASD look less at social stimuli than nonsocial stimuli [36, 51, 52], suggesting that children with ASD shift their gaze to regulate overstimulating social information. Viewing faces and engaging with social partners requires complex timing and attunement, which may be effortful for children with ASD [53]. Together, these findings suggest that increased head movement in reaction to social stimuli may reflect increased sensitivity to social scenes among children with ASD.

Children with ASD may engage in more extreme and quicker head movement than children without ASD because they are unable to regulate incoming social information. Possible disruptions in motor planning and head movements early in development may have cascading effects in later social engagement [54, 55]. Given early associations between motor experience and the development of social behaviors [56], early disruptions in head movement may index atypical developmental trajectories [6, 57].

### Limitations and future directions

Differences between children with and without ASD in head displacement and velocity were obtained in a small sample, highlighting the need for replication. The current study tested specific *a priori* hypotheses regarding head movement dynamic differences by nonsocial and social stimuli. Future research could build upon this research and explore whether head movement dynamics varies proportionally as a result of the degree of sociality of the stimulus. Future research with larger sample sizes and a fully counterbalanced protocol will allow researchers to examine more nuanced research questions.

While use of automated measurement marks progress in objectively quantifying head movement dynamics, there were limitations associated with this approach. The inability of the automated software to track extreme head movement and self-occlusion resulted in missing data (~ 17%). Although missing data did not vary by group, the presence of missing data necessitated using epochs of continuous data collection as a unit of analysis. Moreover, although not significant, children with ASD tended to have more epochs of briefer duration than children without ASD. It is possible that an inability to quantify head movement between epochs yielded a conservative assessment of group differences.

Angular displacement and velocity of pitch, yaw, and roll were moderately correlated in our data, and we chose to examine these dynamics separately. An alternative approach could be to model these movements together to assess differences in children with and without ASD. The addition of postural adjustments and muscle tension measurements to the model would allow for examination of coupling between head, neck, and torso in human movement, and potential differences in coupling associated with ASD.

## Conclusions

Using automated measurement, we quantified differences in the quantity and speed of head movement between children with and without ASD, finding differences in the lateral (yaw and roll) but not vertical (pitch) domain. Children with ASD had greater yaw angular displacement and greater yaw and roll angular velocity, and these differences were most pronounced during social stimulus presentation. The results are consistent with the hypothesis that children with ASD use head movement to regulate their direct exposure to potentially arousing social situations. The study reports on a promising advance in objectively characterizing head movement dynamics. Our findings highlight the possibility of using automated measurement of head motion to supplement current diagnostic approaches for ASD. Automated measurement of head motion in varied contexts could provide an objective method of differentiating children with and without ASD. In contrast to previous approaches to head movement quantification, the computer-vision based approach we used here is non-invasive, may be applied to already collected video of children, and may be well suited for use in monitoring change over the course of the disorder and in response to interventions. 

## Additional file

Additional file 1: Summary of Supplementary Data Analyses. (DOCX 23 kb)

## Abbreviations

ANOVA: Analyses of variance
ASD: Autism spectrum disorder
nRMS: Normalized root mean square
RMS: Root mean square
