# Supplementary material for: Objective measurement of head movement differences in children with and without autism spectrum disorder
Source: Mol Autism. 2018 Feb 27;9:14. doi: 10.1186/s13229-018-0198-4 (PMC5828311; doi:10.1186/s13229-018-0198-4)
Supplement: Supplementary file 1 — Summary of Supplementary Data Analyses. (DOCX 23 kb) [file 13229_2018_198_MOESM1_ESM.docx]

**Supplementary Material**

**Methods.**

Mean of Absolute Values. The means of absolute values of pitch, yaw, and roll were used to calculate overall (non-directional) mean values of displacement, indexing average orientation.

(A) Mean of absolute values = $\frac{\left| x_{1} \right|+\left| x_{2} \right|+\ldots+ \left| x_{n} \right|}{n}$

where x_1_…x_n_ are the absolute values of pitch, yaw, or roll for consecutive frames within epoch.

*Peak displacement.* The maximum absolute value within a block was obtained for angular displacement for pitch, yaw, and roll.

*Peak velocity.* The maximum absolute value within a block was obtained for angular velocity for pitch, yaw, and roll.

*Peak scalar velocity.* The time trajectories of the angular displacement tracks of the three rotation angles were transformed to a sequence of direction cosine matrix. Then the time X, Y and Z locations of the noise point of the head were calculated (assuming a head radius of 8cm). The square root of the sum of the squares of the location changes in X, Y and Z directions between two successive video frames (distance traveled) was then divided by the frame interval to determine scalar velocity. The maximum value of the velocity within a block is measured as the peak velocity.

*Acceleration/Deceleration phases.* The number of acceleration and deceleration phases were calculated as the number of zero-crosses of the smoothed acceleration track (the change point between acceleration and deceleration). The smoothing kernel length was 500 video frames (16.7 seconds), which excluded high frequency noise.

**Analysis Plan.**

Separate 2 (Group) by 6 (Video) repeated measures ANOVA were conducted for absolute value of angular displacement, peak displacement, peak velocity, peak scalar velocity, and number of acceleration/deceleration phases for pitch, yaw, and roll.

**Results.**

Full results are contained in Supplementary Table 1.

*Mean of Absolute Values.* There were no significant differences in the absolute values of angular displacement for pitch, yaw, and roll, which index the overall (non-directional) orientation of head position.

*Peak angular displacement.* Children with ASD displayed higher peak angular displacement of yaw and roll than children without ASD, F(1, 39) = 8.74, *p*<.01, n_p_^2^=.20 and F(1, 39) = 5.31, *p*=.03, n_p_^2^=.13, respectively. No group differences were evident for pitch and yaw.

*Peak angular velocity.* Children with ASD had higher peak angular velocities of yaw and roll than children without ASD, F(1, 39) = 8.15, *p*<.01, n_p_^2^=.19 and F(1, 39) = 6.73, *p*=.01, n_p_^2^=.16, respectively. No group differences were evident for peak angular velocity of pitch.

*Peak scalar velocity.* Children with ASD had a higher velocity peak of head movement than children without ASD, *p*<.01, n_p_^2^ =.17.

*Acceleration/Deceleration phases.* No group differences were evident for the number of acceleration and deceleration phases, F(1, 39)= 2.15, *p*=.15, n_p_^2^=.05.

| Table S1. Summary of Supplementary Data Analyses | | | | | | |
| --- | --- | --- | --- | --- | --- | --- |
|  |  | Marginal Mean  Children with ASD | Marginal Mean  Children without ASD | F | *p* | n_p_^2^ |
| Mean of Absolute Values  (radians) | Pitch | .34 | .31 | .29 | .60 | <.01 |
|  | Yaw | .10 | .09 | .81 | .37 | .02 |
|  | Roll | .10 | .08 | 3.2 | .08 | .08 |
|  |  |  |  |  |  |  |
| Peak Angular Displacement  (radians) | Pitch | .40 | .34 | 2.86 | .10 | .07 |
|  | Yaw | .36 | .26 | 8.74 | <.01* | .20 |
|  | Roll | .32 | .24 | 5.31 | .03* | .13 |
|  |  |  |  |  |  |  |
| Peak Angular Velocity  (radians/second) | Pitch | .14 | .12 | 3.21 | .08 | .08 |
|  | Yaw | .11 | .08 | 8.15 | <.01* | .19 |
|  | Roll | .80 | .60 | 6.73 | .01* | .16 |
|  |  |  |  |  |  |  |
| Peak Scalar Velocity  (radians/second) |  | .32 | .26 | 8.3 | <.01* | .17 |
|  |  |  |  |  |  |  |
| Acceleration/  Deceleration phases |  | 115.90 | 172.23 | 2.15 | .15 | .05 |
